# Abnormal Parietal Brain Function in ADHD: Replication and Extension of Previous EEG Beta Asymmetry Findings

**DOI:** 10.3389/fpsyt.2014.00087

**Published:** 2014-07-24

**Authors:** T. Sigi Hale, Andrea M. Kane, Kelly L. Tung, Olivia Kaminsky, James J. McGough, Grant Hanada, Sandra K. Loo

**Affiliations:** ^1^Department of Psychiatry and Biobehavioral Sciences, UCLA Semel Institute for Neuroscience and Human Behavior, Los Angeles, CA, USA

**Keywords:** ADHD, parietal, asymmetry, laterality, information processing, EEG, beta, TPJ

## Abstract

**Background:** Abundant work indicates ADHD abnormal posterior brain structure and function, including abnormal structural and functional asymmetries and reduced corpus callosum size. However, this literature has attracted considerably less research interest than fronto-striatal findings.

**Objective:** To help address this imbalance, the current study replicates and extends our previous work showing abnormal parietal brain function in ADHD adults during the Conner’s Continuous Performance Test (CPT).

**Method:** Our previous study found that ADHD adults had increased rightward EEG beta (16–21 Hz) asymmetry in inferior parietal brain regions during the CPT (*p* = 0.00001), and that this metric exhibited a lack of normal correlation (i.e., observed in controls) with beta asymmetry at temporal–parietal regions. We re-tested these effects in a new ADHD sample and with both new and old samples combined. We additionally examined: (a) EEG asymmetry in multiple frequency bands, (b) unilateral effects for all asymmetry findings, and (c) the association between EEG asymmetry and a battery of cognitive tests.

**Results:** We replicated our original findings by demonstrating abnormal rightward inferior parietal beta asymmetry in adults with ADHD during the CPT, and again this metric exhibited abnormal reduced correlation to temporal–parietal beta asymmetry. Novel analyses also demonstrated a broader pattern of rightward beta and theta asymmetry across inferior, superior, and temporal–parietal brain regions, and showed that rightward parietal asymmetry in ADHD was atypically associated with multiple cognitive tests.

**Conclusion:** Abnormal increased rightward parietal EEG beta asymmetry is an important feature of ADHD. We speculate that this phenotype may occur with any form of impaired capacity for top-down task-directed control over sensory encoding functions, and that it may reflect associated increase of attentional shifting and compensatory sustained/selective attention.

## Introduction

If a person wants to find an apple “to eat” on a cluttered countertop, it is task-adaptive to identify that stimulus using the minimal sensory detail required. Here, the apple’s constituent esthetic characteristics and/or peripheral information are task-extraneous. Alternatively, if an artist wants to paint a still-life portrait of said apple, they should indulge as much sensory detail as possible. One approach seeks to *identify a stimulus* using the minimal sensory detail required. The other seeks to indulge as much detail as possible in order to generate a prolonged *sensory immersive experience*.

Our current line of research began with the simple precept that the first of these approaches (“to identify a stimulus”) is critical for task-directed actions, and that mobilizing this task-specialized manner of information processing likely depended on the coordinated functioning of multiple distributed brain systems, such as: (1) verbal working memory (VWM) to sequence, direct, maintain, and update task directives [with possible support from spatial working memory (SWM) to model integrated plan steps] (1–4), (2) SWM to generate predictive sensory models to help bias downstream processing toward task stimuli (5, 6), (3) fast perceptual identification of task-relevant content (7, 8), and (4) translation of that identified content into verbal articulatory codes that can be readily integrated with, and used to update, task-plans in VWM (9).

We hypothesized that the coordinated functioning of such elements formed an emergent task-directed brain-system or neural context that optimized multiple distributed brain functions toward task-directed actions (i.e., a task-directed neurocognitive network) (10, 11). We surmised that any impairment to this system, no matter the cause, should result in less efficient task-directed (top-down) control over sensory encoding, with an associated increased exposure to off-task sensory content. More specifically, we expected this circumstance to result in a greater proportion of off-task content being perceptually engaged per incident of task-adaptive identification and verbal encoding of task-relevant stimuli, and that this would be indexed by an increased weighting of right hemisphere (RH) biased visuo-perceptual versus left hemisphere (LH) biased verbal sensory encoding during task challenges (11).

We first examined this hypothesis in ADHD adults using behavioral laterality paradigms. These demonstrated: an ADHD bias toward non-verbal sensory encoding, greater RH contribution to processing task stimuli, associated linguistic impairments, and abnormal interhemispheric interaction (12, 13), and further specified that this pattern could be modulated via top-down attentional resources (13), bore advantages for RH specialized abilities (13), and impacted high-order cognitive functions (14). Then, using fMRI and EEG we found that RH bias in ADHD was mainly evident during sub-executive operations (15), and that it exhibited: a unique developmental course among families heavily loaded for non-persistent ADHD (16), stronger expression with greater ADHD family loading (16), and stronger expression among carriers of the DRD4–7 repeat allele and other ADHD risk factors (unpublished). Finally, a robust and literature-consistent (17, 18) biomarker was identified. ADHD subjects showed highly significant rightward EEG high-beta (16–21 Hz) asymmetry in inferior parietal brain regions during the Conners’ Continuous Performance Test (CPT) (19).

Although not yet widely understood, these findings are well aligned with extant ADHD literature. Slow naming speed is identified in ADHD (20–27), which is consistent with impoverished LH relative to RH contribution to sensory processing. Previous behavioral laterality studies of ADHD have also indicated increased RH contribution (28, 29). Functional imaging studies at rest or during simple (i.e., sub-executive) challenges have shown a pattern of reduced LH (30–33), and/or increased RH contribution (15, 34–37), and recent diffusion tensor imaging studies have reported greater RH parietal (38) and frontal (39) fractional anisotropy in ADHD. Moreover, a lack of normally occurring L > R asymmetry in prefrontal cortical convolution complexity has been reported (40), as well as increased RH visual cortex volumes (41). Finally, identified abnormal posterior corpus callosum size (42) and function (34, 43–45) clearly implicates some form of abnormal integration of verbal and non-verbal sensory operations in ADHD.

With complex executive function (EF) tasks the literature is more variable, showing diffuse effects mainly consistent with variable weaknesses across multiple brain systems (46–49). Nevertheless, several studies have shown a greater association between ADHD behavioral performance and right-sided brain structure and/or function (50–57), and EEG studies that directly examined activation asymmetry and/or left-RH differences have consistently shown an R > L pattern in posterior brain regions (16–19, 34, 35, 37). Finally, a recent meta-analysis of ADHD functional imaging reported hyper-activation of the strongly right-lateralized ventral attention network (VAN), noting it may be related to increased bottom-up visuo-perceptual processing of task-extraneous stimuli (58); and consistent with this Fassbender and Schweitzer (59), via an earlier review of ADHD brain imaging literature, also suggested that ADHD involves an increased reliance on neuroanatomy associated with visual/spatial and motoric (versus verbal) processing during task operations.

Together, our studies and the above literature, implicate some form of abnormal increased weighting of RH non-verbal processing in ADHD. However, our conceptual framework suggests this phenotype may not be “ADHD specific” *per se*, but rather more broadly reflective of any form of impaired task-directed brain-system functioning. Consistent with this, rightward biased brain function has been reported across multiple circumstances linked to attention difficulties, and that are often comorbid with ADHD (e.g., anxiety, depression, sleep deprivation, novelty seeking, reading disability, etc.) (60–70). Accordingly, a primary challenge for our current line of research is to try to identify the specific form (or forms) of rightward biased processing and associated putative task-directed brain-system impairments that most commonly underlie ADHD.

To pursue this goal, we previously evaluated whether rightward biased information processing in ADHD was distinct from the similarly described characteristic in reading impaired samples (65). At that time, increased RH parietal EEG beta activity had been identified in dyslexia, sans attention impairment [for review see Ref. (71)], and in ADHD children, sans reading impairment (17). However, this outcome had not yet been established in ADHD adults. Our study filled this gap by showing that adults with ADHD had highly significant rightward beta (16–21 Hz) inferior parietal EEG asymmetry during the Conners’ CPT, which could not be attributed to poor linguistic ability (19). This study also demonstrated that this characteristic was: (a) specific to the beta frequency band, (b) not normally associated (i.e., observed in controls) with immediately anterior temporal–parietal beta asymmetry, and (c) linked to better CPT performance. The beta band specificity of these effects supported the view that rightward parietal EEG asymmetry in ADHD was linked to some form of abnormal attention-directed information processing [for review, see Ref. (72–74)] that is sensitive to transitions between verbal and non-verbal sensory encoding functions (75, 76).

The current study’s goal was to replicate these EEG beta findings in a larger ADHD sample and extend these results by newly evaluating: (a) EEG asymmetry across multiple frequency bands and brain regions, (b) whether asymmetry in ADHD can be attributed to unilateral activations, (c) the correlations among asymmetry effects, and (d) the associations between EEG asymmetry and cognitive ability. This last goal is critical in that, as noted, our conceptual framework suggests that increased RH contribution to sensory processing could result from multiple and variable impairments across a set of integrated brain functions that serve task-directed actions. By examining the association between sensory processing asymmetry in ADHD and a battery of cognitive tasks, we hope to gain insight into what aspects of putative task-directed brain-system functioning might be most proximal to manifest rightward biased information processing in ADHD. These study goals are summarized in Table 1.

**Table 1:** **Summary of study goals**.

**FINDINGS TO REPLICATE IN CURRENT STUDY (19)**
Adults with ADHD exhibited increased rightward EEG beta2 (16–21 Hz) asymmetry in inferior parietal brain regions (P8–P7 asymmetry index) during the CPT compared to controls (*p* = 0.00001) ADHD rightward asymmetry in this region was only evident in the beta2 frequency band While beta2 asymmetry at inferior parietal and temporal-parietal regions were highly (positively) correlated in controls (*p* < 0.00001), they were not associated in ADHD subjects (*p* = 0.49) ADHD subjects had increased CPT commission errors (*p* = 0.025), and rightward beta2 P8–P7 asymmetry was correlated with making fewer such errors (*p* = 0.048)
**NEW ASSESSMENTS**
Examine asymmetry across multiple frequency bands and brain regions Examine whether asymmetry effects are attributable to unilateral activations Examine associations among uncovered asymmetry effects Examine associations between uncovered asymmetry effects and cognitive abilities

## Materials and Methods

### Study sample

All subjects for the current study were participants in a previous UCLA ADHD family genetics study (77, 78). Our original published work (19) that the current study replicates and extends, also derived its sample from this data set, but at an earlier stage (i.e., when there was a smaller sample). Participation in this UCLA ADHD Genetics study required that families had at least two ADHD affected offspring. Hence, all subjects in the previous and the current study (cases and controls) are the biological parents of children with ADHD.

After receiving verbal and written explanations of study requirements, participants provided written informed consent approved by the UCLA Institutional Review Board. Through the UCLA ADHD Genetics Study, all subjects were screened for ADHD and other psychiatric disorders via direct interviews using the Schedule for Affective Disorders and Schizophrenia-Lifetime Version [SADS-LAR; (79)] supplemented with the Behavioral Disorders supplement from the Schedule for Affective Disorders and Schizophrenia for school aged children-Present and Lifetime Version [KSADS-PL; (80)]. All interviews were conducted by clinical psychologists or highly trained interviewers with extensive experience in psychiatric diagnoses. “Best estimate” diagnoses were determined after individual review of diagnoses, symptoms, and impairment level by senior clinicians (81). Inter-rater reliabilities were computed with a mean weighted kappa of 0.84 across all diagnoses with a >5% occurrence in the sample.

Handedness was assessed with a shortened version of the Edinburgh Handedness Inventory (82). This handedness inventory uses seven questions regarding hand preference and produces scores ranging from −14 (indicating maximum left handedness) to 14 (indicating maximum right-handedness). This measures was dichotomized with scores ranging from 8 to 14 indicating “definite right-handedness,” and scores <8 indicating “marginal or non-right-handedness.”

Subjects were excluded based on the following criteria: past or current documented neurological disorder, a significant head injury resulting in loss of consciousness, a diagnosis of schizophrenia, schizophrenia spectrum disorders, autism, or an estimated Full Scale IQ <80. We did not directly assess for language impairment in our sample. However, an assessment of group differences in several linguistic measures was performed to help rule out language impairment in the ADHD sample.

Inclusion criteria for the present study required a lifetime diagnosis of ADHD, and for non-ADHD controls, no evidence of past or current ADHD (i.e., reporting four or fewer ADHD symptoms in childhood and as adults). In our original study, we required a current rather than lifetime diagnosis of ADHD. This change reflects an increased interest in the brain function characteristics of individuals who present with childhood ADHD, regardless of long-term outcomes. Twenty-three of 90 ADHD subjects in the current study met criteria for lifetime ADHD, but not current (i.e., 67 had current). Five ADHD subjects were on stimulant medication and five were on medication for depressive symptoms (SSRIs). The impact of medicated subjects was directly assessed for all reported analyses. See Table 2 for sample demographics.

**Table 2:** **Sample demographics**.

Clinical variables	Controls *N* = 103	ADHD *N* = 90	Statistic
IQ	$\bar{x}=112$, STD = 14.1	$\bar{x}=113.7$, STD = 14.6	*t* = −0.83, *p* = 0.41
Age	$\bar{x}=44.6$, STD = 6	$\bar{x}=44$, STD = 5.7	*t* = 0.88, *p* = 0.38
Sex	50 F, 53 M	48 F, 42 M	χ^2^ = 0.44, *p* = 0.51
ADHD type	N/A	33C, 48I, 9H	N/A
Right-handed	9 NSR, 94 R	13 NSR, 75 R	χ^2^ = 1.7, *p* = 0.19
Anxiety	20 Affected	33 Affected	χ^2^ = 7.4, *p* = 0.006
Mood	5 Affected	15 Affected	χ^2^ = 7.4, *p* = 0.007
Vocabulary	$\bar{x}=12.4$, STD = 2.9	$\bar{x}=12.6$, STD = 3.0	*t* = −0.49, *p* = 0.62
Phonology	$\bar{x}=24.7$, STD = 4.7	$\bar{x}=25$, STD = 3.9	*t* = −0.46, *p* = 0.64
Reading	$\bar{x}=50.1$, STD = 3.8	$\bar{x}=50.2$, STD = 4.3	*t* = −0.16, *p* = 0.87
Spelling	$\bar{x}=44$, STD = 5.2	$\bar{x}=43.5$, STD = 5.4	*t* = 0.49, *p* = 0.63
On meds	None	5 Stim., 5 Dep.	N/A

***Estimated full IQ**, estimated from block-design and vocabulary subtest of WAIS-III; **ADHD type**: C, combined; I, inattentive; H, hyperactive (lifetime diagnosis); **NSR**, not strong right-handed; **R**, right-handed; **χ^2^**, chi-square test; **anxiety/mood** reflect definite diagnosis of at least one current anxiety and/or mood disorder as assessed by direct interview using SADS-LAR (see text for reference); see Table 7 for description of linguistic measures; **Stim**., stimulant medications for ADHD; **Dep**., medication for depression*.

### Procedures

Typical testing procedures for the UCLA ADHD genetics study involved a mother and/or father and two ADHD affected offspring coming to UCLA for single visit (although fathers were often tested on a separate day). During the visit, each family member underwent a clinical, cognitive, and EEG testing battery, with the order of delivery of each component determined by logistical considerations. However, during EEG testing the Conners’ CPT (83) was always delivered first, followed by additional conditions that are not reported. Recordings were performed in a small private room with a sole male technician administering the protocol.

### Electrophysiological measures

EEG recording (256 samples/s) was carried out using 40 silver chloride electrodes using the International 10/20 locations and was referenced to an average of signals recorded separately at each ear lobe. Eye movements were monitored by electrodes placed on the outer canthus of each eye for horizontal movements and above the eye for vertical eye movements. EEG was recorded during the Conners’ CPT II (83), lasting for 15 min. Continuous EEG data were subjected to mean removal, a band pass filter (including data between 0.6 and 59 Hz), and automatic artifact detection via MANSCAN software (SAM Technology, Inc., San Francisco, CA, USA http://www.manscaneeg.com) designed to identify dead and bad channels, vertical and horizontal eye movements, saturation, muscle and movement artifact, and line frequency noise. Subsequent to this automated procedure, an experienced EEG technician visually inspected all data and identified any residual contaminants. Next, continuous EEG was broken into 1-s epochs and artifact-containing epochs were removed on a channel specific basis. Remaining artifact free epochs were then Fast Fourier Transformed (FFT) using *MANSCAN EEG software*, which uses a Welch’s Periodogram approach (84). We specified 1-s data segments with 50% overlap, and a Hanning Windowing function to generate spectral content at a 1 Hz resolution. Spectral data were then averaged and EEG power (mv^2^) from 1 to 21 Hz was exported in 1 Hz bins (e.g., 0–1, 1–2, …, 20–21). Absolute power between 1–4 Hz (Delta), 4–8 Hz (Theta), 8–10 Hz (alpha1), 10–12 Hz (alpha2), 12–16 Hz (beta1), and 16–21 Hz (beta2) frequencies was averaged for each electrode. These bands are non-overlapping. For example, beta1 extends up to the 15–16 1-Hz bin, while beta2 begins at the 16–17 1-Hz bin. Technicians involved in the EEG recording and processing were blind to ADHD diagnostic status.

Our primary interest was to replicate and extend our previous finding of R > L beta2 asymmetry in the inferior parietal region of adults with ADHD during the CPT (19). Thus, the current study assessed EEG power asymmetry in adults with ADHD and controls. Asymmetry indices (AIs) were generated for nine homologous right-left electrode pairs (AF4–AF3, F4–F3, F8–F7, FT8–FT7, T8–T7, TP8–TP7, P4–P3, P8–P7, O2–O1) using the following standard calculation: [(R − L)/(R + L) × 1000].

Lastly, in our efforts to further characterize the nature of atypical functional asymmetry in ADHD, the current study also examined separate left and RH activations for AIs that showed significant group differences. To generate EEG power measures for individual electrodes and frequency bands of interest, power in a target frequency at a given electrode was divided by the averaged total power (1–21 Hz) across the scalp (26 electrodes in standard 10–20 positions). This electrode-set did not include electrodes placed inferiorly to the axial plane of the 8/7s or electrodes used for detecting eye artifact (FP1, FPZ, FP2, F9, F10). These individual electrode measures will be referred to as “globally normalized” (GN) power.

### The CPT task

The CPT required subjects to monitor a central fixation on a computer screen while single capital letters are sequentially and centrally presented during 6 continuous blocks of 20 trials with either 1, 2, or 4 s inter-stimulus intervals (ISIs) (2 blocks for each ISI). Total task time is 15 min. The order of ISI block presentation is randomized within subjects. The task requires subjects to press the space bar using their dominant hand with every letter presentation except for the letter “X.” The “X” occurs on 10% of the trials within a given ISI block. Behavioral performance was assessed using the following standard CPT measures (85): (1) commission errors: a failure to inhibit response when an “x” is presented, (2) omission errors: a failure to respond when any letter other than “x” is presented, (3) hit reaction time: response time for all letters other than “x,” (4) hit reaction time standard error: reaction time variability, (5) response bias: signal detection measure (beta) indicating impulsive versus conservative response styles, (6) sensitivity: signal detection measure (*d*′) indicating accuracy adjusted for false alarms.

## Analyses

### Overview

In the original study we tested beta asymmetry effects; however, as a *post hoc* we also examined CPT P8–P7 asymmetry in multiple frequency bands to test the “beta specificity” of this effect. In the original study we also examined correlations between the beta2 P8–P7 AI (i.e., our effect of interest, or EoI) and other AIs in the beta band, and tested the association between P8–P7 beta2 asymmetry and CPT task performance. The current study builds on these approaches.

### Primary analyses – parts 1 and 2

(1) To examine the robustness of our previously identified rightward beta2 asymmetry in the inferior parietal region (P8–P7) of adults with ADHD during the CPT (i.e., the EoI), we re-tested our original analysis with a new cohort of adult ADHD subjects (*n* = 43) and with our current full sample comprised of both the original and newly added ADHD participants (*n* = 31 + 43 = 74).
(2) Next, in an attempt to further characterize this asymmetry effect, three additional analyses were performed: (a) to assess whether rightward EEG asymmetry in ADHD is specific to the beta2 frequency band at the P8–P7 AI, we examined EEG asymmetry across multiple frequency bands and brain regions, (b) to examine whether uncovered asymmetry effects in ADHD were driven by separate left and/or RH activations, we tested group differences in unilateral right and LH electrodes comprising any AI that showed a group difference, and (c) to further examine whether the EoI was distinct, or associated with other asymmetry effects, we tested correlations between the EoI and any newly uncovered asymmetry findings.

These analyses provided an opportunity to directly re-assess two key outcomes from our original study: (1) group differences at the P8–P7 AI were only evident for the beta2 frequency band, and (2) beta2 asymmetry at P8–P7 and TP8–TP7 AIs were highly correlated in controls (*p* < 0.00001), but not in ADHD subjects (*p* = 0.49) (group differences in correlation values were tested with Fisher’s *r*-to-*z* test: *z* = 3.35, *p* = 0.0004).

### Secondary analyses

Secondary analyses examined whether EEG asymmetries in ADHD subjects predicted cognitive abilities (using an expanded cognitive battery). For each AI that showed group asymmetry differences, linear regression was used to examine whether EEG asymmetry interacted with ADHD diagnostic status to predict cognitive abilities. Group differences in cognitive abilities were also directly assessed.

These analyses allowed us to re-assess two key outcomes from our previous study: (1) ADHD subjects had increased CPT commission errors (*p* = 0.025) and reduced sensitivity (*d*′: *p* = 0.02), and (2) in ADHD subjects only, greater rightward beta2 asymmetry at the P8–P7 AI was associated with fewer commission errors (*p* = 0.048).

### Statistical approach

All analyses were performed using SPSS (v21). All significant findings were re-tested with medication status (i.e., on/off stimulant medication and on/off depression medication) entered as additional covariates and the resultant medication adjusted *p*-values for reported findings are provided. Due to the highly targeted nature of these replication analyses and our associated *a priori* hypotheses (i.e., rightward parietal asymmetry in ADHD), results are reported without multiple comparison adjustments. Moreover, in our efforts to further characterize the EoI, our interest was specifically to identify meaningful *patterns of EEG-to-behavior* effects. Hence, we present these findings without multiple comparison adjustments and limit our interpretation of results to a “pattern level of analysis,” which helps guard against type-1 error. The three statistical methods utilized in the current study are presented below.

#### Method 1

General linear model univariate ANOVA was utilized to examine group differences in EEG asymmetry and cognitive task performance. Outcome measures were entered as the dependent variable, with diagnostic status (ADHD versus Control) entered as a fixed factor, and handedness and the presence of an anxiety and/or mood disorder were entered as covariates (anxiety and mood showed group differences and handedness approach significance: *p* = 0.11). Moreover, since subjects responded with their dominant hand during the CPT, co-varying for handedness in our EEG analysis also adjusted for any CPT response hand effects.

#### Method 2

Pearson’s correlation analysis was used in control subjects to assess the associations between P8 and P7 beta2 asymmetry (the EoI) and other EEG asymmetries that showed group differences, and partial correlations were used to this same end in ADHD subjects while adjusting for medication status. Where relevant, we used Fisher’s *r*-to-*z* test to statistically examine the difference between two correlations (86). For this test, correlations are first transformed so that they are unbounded using the inverse hyperbolic tangent function. Next, the difference between the transformed correlations is converted to a *Z* score based on the sample sizes and then a *p*-value is obtained based on the *Z* score.

#### Method 3

Linear regression was used in secondary analyses to examine whether ADHD affection-status interacted with EEG asymmetry measures of interest (i.e., that show group differences) to predict cognitive abilities. Here, a cognitive measure was entered as the outcome variable, with the following variables entered as predictors: handedness, anxiety-status, mood-status, affection-status, the AI of interest, and an interaction term of affection-status by the AI of interest. In this way, we assessed whether ADHD subjects showed unique associations between EEG asymmetry and cognitive measures. Prior to performing these analyses, we utilized the univariate procedure described above to characterize group differences on cognitive measures used in these secondary analyses (Table 7).

## Results

### Primary analyses – part 1: Testing the robustness of the effect of interest

#### Demographic data for replication samples

Table 3 shows specific demographic information for our EoI replication analyses (i.e., rightward beta2 asymmetry at the P8–P7 AI in adults with ADHD during the CPT) as well as demographic data associated with our original analyses of this effect. The only notable demographic difference between samples is that the new ADHD cohort did not exhibit greater expression of comorbid anxiety than the controls. However, with all ADHD subjects combined, this effect was significant. Please note that sample sizes reflect the number of subjects contributing useable data to the P8–P7 beta2 AI. The total number of EEG participants in the current study that contributed useable data (i.e., across all AI measures) was larger (see Table 2 for full sample demographics).

**Table 3:** **Demographic information for subjects comprising three analyses of our effect of interest (P8–P7 beta2 asymmetry)**.

Original subjects	Controls *N* = 84	Original ADHD *N* = 31	Stats
IQ	$\bar{x}=113$, STD =14.3	$\bar{x}=110$, STD = 15.5	*t* = 0.9, *p* = 0.40
Age	$\bar{x}=\text{44}.\text{6}$, STD = 6	$\bar{x}=\text{44}.\text{6}$, STD = 6	*t* = 0.01, *p* = 0.99
ADHD type	N/A	5C, 27I, 3H	N/A
Sex	45F, 39M	19F, 12M	χ^2^ = 0.55, *p* = 0.46
Right-handed	6 NSR, 78 R	2 NR, 29 R	fe, *p* = 1
Anxiety	17 Affected	15 Affected	χ^2^ = 8.9, *p* = 0.003
Mood	5 Affected	6 Affected	χ^2^ = 4.7, *p* = 0.03
On meds	None	None	N/A

**New ADHD**	**Controls *N* = 84**	**New ADHD cohort *N* = 43**	**Stats**

IQ	$\bar{x}=113$, STD = 14.3	$\bar{x}=117$, STD = 14	*t* = −1.6, *p* = 0.10
Age	$\bar{x}=\text{44}.\text{6}$, STD = 6	$\bar{x}=\text{43}.3$, STD = 5.8	*t* = 1.2, *p* = 0.24
ADHD type	N/A	19C, 20I, 6H	N/A
Sex	45F, 39M	23F, 22M	χ^2^ = 0.07, *p* = 0.80
Right-handed	6 NSR, 78 R	7 NSR, 36 R	χ^2^ = 2.6, *p* = 0.11
Anxiety	17 Affected	13 Affected	χ^2^ = 1.3, *p* = 0.27
Mood	5 Affected	8 Affected	χ^2^ = 4.5, *p* = 0.03
On meds	None	4 Stim., 4 Dep.	N/A

**Full sample**	**Controls *N* = 84**	**All ADHD *N* = 74**	**Stats**

IQ	$\bar{x}=113$, STD = 14.3	$\bar{x}=114$, STD = 14.6	*t* = −0.61, *p* = 0.54
Age	$\bar{x}=\text{44}.\text{6}$, STD = 6	$\bar{x}=\text{43}.8$, STD = 5.8	*t* = 0.83, *p* = 0.41
ADHD type	N/A	27C, 39I, 8H	N/A
Sex	45F, 39M	42F, 32M	χ^2^ = 0.16, *p* = 0.69
Right-handed	6 NSR, 78 R	9 NSR, 65 R	χ^2^ = 1.1, *p* = 0.28
Anxiety	17 Affected	27 Affected	χ^2^ = 6.1, *p* = 0.01
Mood	5 Affected	13 Affected	χ^2^ = 6.7 *p* = 0.01
On meds	None	4 Stim., 4 Dep.	N/A

***Estimated full IQ**, estimated from block-design and vocabulary subtest of WAIS-III; **ADHD type**: C, combined; I, inattentive; H, hyperactive (lifetime diagnosis); **NSR**, not strong right-handed; **R**, right-handed; **χ^2^**, chi-square test; **fe**, Fisher’s exact test; **anxiety/mood** reflect definite diagnosis of at least one current anxiety and/or mood disorder as assessed by direct interview using SADS-LAR (see text for reference); **Stim**., stimulant medications for ADHD; **Dep**., medication for depression treatment*.

#### Analysis of effect of interest for replication samples

Both replication analyses showed the EoI result to be highly significant and in the same direction as our original finding. However, while the pattern of effects was highly similar across all three analyses, the effect was statistically weaker with the new ADHD sample alone, and adjusting for medication status in this analysis revealed a modestly significant replication effect (*p* = 0.046). With the full sample, the replication of the EoI was highly significant (*p* = 0.00003) and remained so after adjusting for medication status (Table 4).

**Table 4:** **Replication of rightward beta2 asymmetry at the P8–P7 AI in adults with ADHD**.

Analyses	Samples	Controls		ADHD		*f*	*df*	*p*	Partial eta^2^	Meds Adj.	ADHD Asym.
		$\bar{x}$	SE	$\bar{x}$	SE	
Original study	84 Controls 31 ADHD	−78.6	11.4	26.8	19.3	21.2	6.108	**0.00001**	0.16	N/A	Rwrd
New ADHD	84 Controls 43 ADHD	−78.3	13.3	−9.7	18.8	8.6	4.122	**0.004**	0.07	0.046	Rwrd
All ADHD	84 Controls 74 ADHD	−79.8	12.7	2.5	13.6	18.8	4.153	**0.00003**	0.11	0.0002	Rwrd

*Univariate analysis of variance was used to test difference between ADHD and Controls in EEG beta2 asymmetry at the P8–P7 asymmetry index during the CPT, while controlling for handedness, anxiety, and mood. Two new replication analyses are shown, along with our original result from a previous study. **$\bar{x}$**, estimated marginal means (more negative mean values = more leftward asymmetry); **SE**, standard error; **Partial eta^2^**, SPSS estimate of effect size for univariate anova procedure; **Meds Adj**., shows medication status adjusted p-values; **ADHD Asym**., shows direction of ADHD asymmetry relative to controls; **Rwrd**, rightward*.

### Primary analyses – part 2: Expanded analysis of EEG asymmetry

#### Testing AI effects

**Figure 1:**
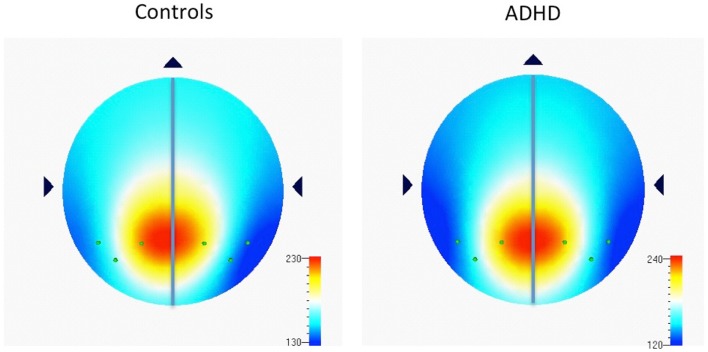
**Distribution of absolute power in beta2 frequency band (16–21 Hz) among parietal electrodes comprising parietal asymmetry indices**. Figure 1 shows EEG data recorded during the CPT, and indicates the spread of beta2 (16–21 Hz) power (μv^2^) among parietal electrodes comprising parietal asymmetry indices (TP7, P7, P3, TP8, P8, P4). Note the leftward distribution of power in controls compared to ADHD.

Examination of multiple frequency bands and AIs across the scalp demonstrated several instances of atypical rightward parietal asymmetry in ADHD. Rightward parietal asymmetry in ADHD subjects was evident at the temporal–parietal AI (TP8–TP7) in theta, beta1, and beta2 frequency bands, the inferior parietal AI (P8–P7) in the beta2 frequency band, and the superior-parietal AI (P4–P3) in theta and beta2 frequency bands (Table 5; Figure 1). As in our previous study, the P8–P7 AI only showed ADHD/control group differences in the beta2 frequency band. There were no significant effects showing leftward parietal asymmetry in ADHD. However, a trend effect indicated ADHD leftward *frontal* asymmetry (F8–F7) in the beta2 band, which is reported due to a conceptual interest.

**Table 5:** **Additional asymmetry effects in ADHD adults during the CPT**.

Laterality index	Controls		ADHD		*f*	*df*	*p*	Partial eta^2^	Meds Adj.	ADHD Asym.	Unilat. Eff.	
	$\bar{x}$	SE	$\bar{x}$	SE							Dir.	*p*
Beta2 F8–F7	41.5	15.2	−3	16.2	3.9	4.161	**0.051**	0.02	0.056	Lwrd		
Theta TP8–TP7	−39.2	12.1	11	12.6	8	4.143	**0.006**	0.05	0.004	Rwrd		
Beta2 TP8–TP7	−64.3	18.8	10.1	19.5	7.3	4.146	**0.008**	0.05	0.017	Rwrd		
Beta1 TP8–TP7	−29.5	16.8	21	17.3	4.2	4.146	**0.043**	0.03	0.046	Rwrd	R ↑	0.05
Beta2 P8–P7	−79.8	12.7	2.5	13.6	18.8	4.153	**0.00003**	0.11	0.0002	Rwrd	R ↑	0.02
Theta P4–P3	−19.1	5.3	6.4	5.7	10.3	4.164	**0.002**	0.06	0.003	Rwrd		
Beta2 P4–P3	−32.5	7.2	−7.8	7.7	5.3	4.163	**0.023**	0.03	0.063	Rwrd	R ↑	0.007

*Univariate analysis of variance was used to test difference between ADHD and controls in EEG asymmetry across multiple frequency bands and locations, while controlling for handedness, anxiety, and mood. **$\bar{x}$**, estimated marginal means (more negative mean values = more leftward asymmetry); **partial eta^2^**, SPSS estimate of effect size for univariate ANOVA procedure; **Meds Adj**., shows medication status adjusted *p*-values; **ADHD Asym**., shows direction of ADHD asymmetry relative to controls; **Rwrd**, rightward; **Lwrd**, leftward; **Unilat. Eff**., shows three instances where rightward asymmetry in ADHD co-occurs with unilateral right-sided increases of globally normalized (GN) EEG power. Note: P8–P7 Beta2 finding with full ADHD sample shown in Table 4 is also shown here*.

#### Unilateral effects for significant AIs

Examination of unilateral effects for AI that showed significant group differences demonstrated that three of seven AI results involved significantly greater right-sided power (Table 5).

#### Testing association among AIs that showed group differences in asymmetry

We used correlation analysis in controls and partial correlations in ADHD subjects (to control for medication status) to assess association between the EoI and the other AIs that showed group differences. The Fisher *r*-to-*z* test was used to assess group differences in correlation effects.

Assessment of correlations between the EoI and the other AIs examined replicated our previous finding. ADHD subjects had a significantly weaker correlation between P8–P7 beta2 asymmetry and TP8–TP7 beta2 asymmetry (Fisher’s *r*-to-*z* test: *z* = 2.2, *p* = 0.03) (Table 6). This was the only group difference in correlation values identified by the Fisher’s *r*-to-*z* test. Note: there were no associations (*r* < 0.02) between the EoI and the frontal trend effect in either group (results not shown).

**Table 6:** **Correlations between the EoI and other AIs that showed rightward asymmetry in ADHD**.

		TP8–TP7			P4–P3	
Effect of interest		Theta	Beta1	Beta2	Theta	Beta2
C: P8–P7 Beta2	r	0.21	0.50	**0.56**	0.17	0.46
	p	0.07	0.000003	**0.00001**	0.13	0.000001
A: P8–P7 Beta2	r	0.20	0.28	**0.25**	0.32	0.43
	p	0.10	0.02	**0.04**	0.007	0.0002

*ADHD and control groups Pearson’s correlations between P8–P7 beta2 asymmetry and all additional asymmetry indices that showed greater rightward asymmetry in ADHD subjects; **C**, controls; **A**, ADHD; ADHD results reflect partial correlation adjusted for medication status; bold highlights that these correlations were significantly different between groups according to Fisher’s *r*-to-*z* test (*z* = 2.2, *p* = 0.03)*.

### Secondary analyses – rightward asymmetry association to cognitive metrics

Cognitive measures utilized in these secondary analyses cover several domains, such as: VWM, SWM, EFs, processing speed, attention, verbal phonologic ability, and motor dexterity (Table 7).

**Table 7:** **Cognitive measures**.

Tasks	Measures	Var. name	Description
Wechsler Intelligence Scale for Adults, 3rd Addition (WAIS-III) (87)	Mental arithmetic Digit span forward (max/raw) Digit span backward (max/raw) Spatial span forward (max/raw) Spatial span backward (max/raw) Working memory index Vocabulary	Arith DSF-max/raw DSB-max/raw SSF-max/raw SSB-max/raw WMI Vocab	Mental arithmetic and digit span tap VWM, and WMI is a standard composite score comprised of digit span, arithmetic, and coding subtests
Sternberg Spatial Working Memory Task (88)	Loads 1, 3, 5, 7 (accuracy, RT, RTSD)	SWM L1 (3, 5, 7) (Acc, RT, RTSD)	Delayed match-to-sample visuo-spatial working memory test
Stroop Task (89, 90)	Color naming speed Word naming speed Interference control	St-color St-word St-inter	Color/word processing speed, and Stroop interference control
Trail Making A and B (91)	Trails A Trails B	Trails A Trails B	Speeded visual attention and set-shifting ability
Conners’ Continuous Performance Test II (CPT) (83)	Commissions Omissions Hit reaction time Hit RT standard Deviation Sensitivity, bias	Commiss Omiss Hit RT Hit RTSD *d*′, Beta	Processing speed, sustained visual attention, and response inhibition
Woodcock–Johnson-Revised (WJ-R), Word Attack (92)	Phonologic processing	Phonologic	Speeded reading of nonsense words tests phonologic ability
Wide Range Achievement Test (WRAT): reading, spelling (93)	Reading Spelling	Reading Spelling	Reading and spelling ability
Relative Hand Skill Task (94)	Sum left Sum Right	L.hand (RH) R.hand (LH)	Left/right hand dexterity via speeded box checking task
Time Discrimination	Green Red correct Total correct Mean correct RT Mean correct RTSD Time difference mean	GreenCorrect RedCorrect TotalCorrect MeanCorrRT MeanCorrRTSD Time_Diff_mean	Presents two spatially fixed (L/R) color circles one at a time. Subjects must decide which was “on” for a longer period of time

The goal of these secondary analyses was to examine whether uncovered atypical EEG asymmetry in ADHD impacts cognitive functioning. However, before examining EEG-to-cognition associations, we first tested group differences in cognitive measures using the univariate procedure described above. Several group differences emerged (Table 8). We did not replicate our original study finding of increased CPT commission errors in ADHD (*p* = 0.025).

**Table 8:** **Cognitive effects**.

Cognitive measures	Control		ADHD		*df*	*f*	*p*	Partial eta^2^	Meds Adj.	ADHD effect
	$\bar{x}$	SE	$\bar{x}$	SE	
CPT hit RTSD	126.7	7.1	151	7.9	4.93	5.1	0.027	0.05	0.034	Variable
SWM L1 RT	942.1	21	1028	23	4.134	7.2	0.008	0.05	0.017	Slower
SWM L1 RTSD	236.1	11.4	277.2	12.6	4.134	5.5	0.02	0.04	0.052	Variable
SWM L3 RT	1084	22.7	1174	25	4.134	6.8	0.01	0.05	0.019	Slower
SWM L5 RT	1187	24	1283	26.2	4.134	7	0.009	0.05	0.014	Slower
SWM L7 RT	1213	26	1328	28	4.134	8.6	0.004	0.06	0.009	Slower
DSB_max	5.0	0.13	5.42	0.15	4.177	3.94	0.049	0.02	0.062	Better

*Univariate analysis of variance was used to test ADHD/controls group differences in cognitive abilities, while controlling for handedness, anxiety, and mood. **$\bar{x}$**, estimated marginal means; **partial eta^2^**, SPSS estimate of effect size for univariate ANOVA procedure; **Meds Adj**., shows medication status adjusted *p*-values; see Table 7 for description of cognitive measures*.

Regression analysis (described above) examined whether ADHD affection-status interacted with EEG asymmetry measures to predict ADHD cognitive characteristics. Several findings demonstrated a unique EEG asymmetry-to-cognition association pattern among ADHD subjects (Table 9). Our previous study’s correlation result indicating an association between greater rightward beta2 asymmetry at the P8–P7 AI and fewer CPT commission errors was not replicated. To help aid the interpretation of significant group × AI interaction effects predicting cognitive measures, correlations between AIs and cognitive measures are shown for each group. Note: positive AI values mean rightward asymmetry and all reported findings from the current study are also summarized below in Table 10.

**Table 9:** **Parietal EEG asymmetry × ADHD status predicting cognitive abilities**.

Diag. × EEG asymmetry predicts	*df*	sb	*t*	*p*	Meds Adj.	EEG-behav. corr. (*r*-vals) and ADHD eff.			Diag. × EEG asymmetry predicts	*df*	sb	*t*	*p*	Meds Adj.	EEG-behav. corr. (*r*-vals) and ADHD eff.		
**F8–F7 beta2**						*r*(*C*)	*r*(*A*)	e	**TP8–TP7 beta2**						*r*(*C*)	*r*(*A*)	e
*VWM*									*VWM*								
Arithmetic	6.151	0.20	2.0	**0.045**	0.047	0.01	0.33*	b	DS-overall	6.138	0.29	2.3	**0.02**	0.016	− 0.20^t^	0.18	b
*SWM*									DSF-raw	6.138	0.27	2.2	**0.03**	0.022	− 0.19	0.15	b
SSF-raw	6.147	0.21	2.2	**0.028**	0.03	−0.05	0.28^+^	b	DSF-max	6.138	0.27	2.2	**0.03**	0.021	− 0.19	0.14	b
*CPT*									DSB-raw	6.138	0.30	2.4	**0.016**	0.013	− 0.18	0.19	b
Hit RT	6.81	−0.30	−2.1	**0.036**	0.02	0.29^+^	−0.19	b	DSB-max	6.138	0.27	2.2	**0.03**	0.03	− 0.14	0.19	b
**TP8–TP7 Theta**									WM index	6.138	0.31	2.5	**0.014**	0.01	− 0.15	0.28^+^	b
*SWM*									*WJ-R*								
L5 RTSD	6.106	0.25	2.0	**0.046**	0.045	−0.18	0.10	w	Phonologic	6.126	0.29	2.2	**0.027**	0.025	− 0.12	0.26^+^	b
*Time disk*									*Box check*								
Green Corr.	6.94	−0.26	−2.1	**0.04**	0.02	0.28^+^	−0.17	w	R > L Diff.	5.116	0.40	2.9	**0.004**	0.005	−0.39*	0.11	–
**TP8–TP7 beta1**									R.hand (LH)	5.116	0.35	2.6	**0.01**	0.02	− 0.06	0.43*	–
*VWM*									*Time disk*								
DS-overall	6.138	0.35	3.0	**0.003**	0.003	−0.30*	0.16	b	Total Corr.	6.95	− 0.29	− 2.0	**0.044**	0.04	0.16	− 0.18	w
DSF-raw	6.138	0.36	3.2	**0.002**	0.002	−0.31*	0.19	b	**P8–P7 beta2**								
DSF-max	6.138	0.38	3.4	**0.001**	0.001	−0.33*	0.20^t^	b	*Time disk*								
DSB-raw	6.138	0.32	2.8	**0.005**	0.005	−0.23^+^	0.24^+^	b	Total Corr.	6.96	− 0.31	− 2.2	**0.028**	0.016	0.24^t^	− 0.16	w
DSB-max	6.138	0.28	2.4	**0.016**	0.016	−0.20^t^	0.21^t^	b	**P4–P3 Theta**								
WM index	6.138	0.32	2.8	**0.007**	0.007	−0.24^+^	0.18	b	Trails								
*SWM*									Trails A	6.155	0.23	2.3	**0.024**	0.017	− 0.11	0.21^t^	w
L5 Acc.	6.107	−0.25	−2.3	**0.026**	0.03	0.14	−0.26^+^	w	Trails B	6.155	0.23	2.3	**0.023**	0.022	− 0.19^t^	0.13	w
*WJ-R*									*Time disk*								
Phonologic	6.126	0.27	2.3	**0.024**	0.025	−0.19	0.19	b	Green Corr.	6.108	− 0.31	− 3.0	**0.003**	0.003	0.12	− 0.31^+^	w
*Box check*									**P4–P3 beta2**								
R > L Diff.	5.116	0.32	2.6	**0.01**	0.01	−0.36*	0.12	–	*Trails*								
									Trails A	6.154	0.21	2.0	**0.05**	0.028	− 0.04	0.26^+^	w
									Trails B	6.154	0.22	2.1	**0.04**	0.025	0.02	0.28^+^	w

Linear regression analysis was used to examine the interaction effects of ADHD diagnostic status and parietal EEG asymmetry on subjects’ cognitive abilities. Each test was adjusted for the effects of handedness, anxiety, and mood. **sb**, standardized beta; **Meds Adj**., shows medication status adjusted *p*-values; **EEG-behav. corr (*r*-values) and ADHD effect**, first two columns show *r*-values for correlations between EEG asymmetry and cognitive measure – ***r*(C)**, controls; ***r*(A)**, ADHD; ^**+**^, significant (*p* < 0.05); *, significant (*p* < 0.01); **^t^**, trend (*p* < 0.10); **e**, effect, this column shows “**b**” or “**w**” indicating rightward asymmetry in ADHD subjects predicted “better” or “worse” behavioral outcomes; **raw**, digit and spatial span standard performance score; **max**, digit and spatial span maximum span length achieved; see Table 7 for description of cognitive measures.

**Table 10:** **Results summary**.

Re-tested original findings	Rep.	ADHD outcome
Increased rightward EEG beta2 asymmetry in ADHD	Yes	This result replicated in our full sample (with new and original ADHD subjects). With new ADHD subjects only, it replicated (*p* = 0.004), but adjustment for medication status revealed a modest statistical effect (*p* = 0.046)
Group differences at P8–P7 AI were only evident in beta2 frequency band	Yes	Replicated
Beta2 asymmetry at P8–P7 and TP8–TP7 AIs were highly correlated in controls, but not in ADHD subjects	Yes	Replicated
In ADHD subjects only, rightward beta2 P8–P7 asymmetry was associated with fewer commissions	No	Not Replicated – in our full sample ADHD subjects did not show significant deficit for commissions, nor was EEG asymmetry associated with commissions
**NEW ANALYSES**		
Examine asymmetry across multiple frequency bands	New	Rightward asymmetry was evident for all parietal measures (TP8–TP7, P8–P7, P4–P3). TP8–TP7 effects occurred in theta and beta1 and 2. P8–P7 asymmetry was exclusive to beta2. P4–P3 effects occurred in theta and beta2
Examine whether asymmetry effects are driven by unilateral activations	New	Only RH beta showed significant unilateral effects. RH P8 and P4 beta2 power were increased. RH TP8 beta1 power was increased
Examine correlations among uncovered asymmetry effects	New	Compared to controls, ADHD subjects’ P8–P7 beta2 asymmetry exhibited weak association to TP8–TP7 asymmetry in beta1 and beta2 frequencies, but stronger association to P4–P3 asymmetry in theta
Examine associations between uncovered asymmetry effects and cognitive abilities	New	A majority of asymmetry-to-cognitive associations occurred with the TP8–TP7 asymmetry index. These showed mainly positive effects of rightward asymmetry on cognition in beta1 and beta2 frequencies, barring tasks that required fast continuous sensory processing (time disk., trails, SWM), which showed negative effects. P8–P7 and P4–P3 indices showed only negative associations – also for the more sensory weighted tasks

## Discussion

Our original study compared adult ADHD and control subjects’ EEG asymmetry during the Conners’ CPT in beta1 (12–16 Hz) and beta2 (16–21 Hz) frequency bands (19). That study uncovered single highly significant effect (*p* = 0.00001) showing ADHD adults had increased rightward P8–P7 beta2 asymmetry during the CPT, and in *post hoc* analysis confirmed that this result was specific to the beta2 frequency band. We refer to this finding as our “EoI.” That study also found that while controls showed a robust positive correlation between beta2 asymmetry at the P8–P7 and the immediately anterior TP8–TP7 AI, ADHD subjects had no such effect. Lastly, this original study demonstrated a unique ADHD association between rightward P8–P7 beta2 asymmetry and fewer CPT commission errors.

The current study sought to replicate and build upon these results using a larger ADHD sample. Expanded elements included: (a) testing additional frequency bands, (b) testing unilateral effects for all asymmetry findings, and (c) testing the association between ADHD abnormal asymmetry and a battery of cognitive tests. This current study replicated the EoI (*p* = 0.00003), again demonstrating that ADHD adults exhibit atypical rightward P8–P7 beta2 asymmetry during the CPT. Moreover, group differences at this inferior parietal index were again limited to the beta2 frequency band. We also replicated our previous finding showing that ADHD subjects exhibit a reduced correlation between beta2 asymmetry at the P8–P7 and TP8–TP7 AIs. We did not replicate our original finding showing that rightward P8–P7 beta2 asymmetry in ADHD was associated with fewer CPT commission errors.

In addition to these replicated effects, the current study added several new findings. With a larger ADHD sample and using multiple frequency bands, we uncovered a broader pattern of abnormal rightward parietal asymmetry during the CPT. ADHD rightward asymmetry was evident in the beta2 band across all parietal indices (TP8–TP7, P8–P7, P4–P3), and in additional bands for temporal–parietal (TP8–TP7: theta, beta1), and superior-parietal (P4–P3: theta) indices. We also identified that three of five ADHD parietal beta asymmetry findings, including the EoI, were linked to greater unilateral right-sided beta power. Lastly, we uncovered multiple abnormal associations between rightward parietal asymmetry in ADHD and cognitive abilities.

### Lateralized brain function and the parietal lobes

The nature of parietal brain function continues to be debated; however, some general themes have emerged. First, it has become increasingly clear that the complexity of the human parietal cortex mirrors that of the frontal lobes and plays key roles in many of the higher order operations traditionally ascribed to frontal brain regions (95–97). Next, parietal brain function has been broadly associated with processing information in a spatial context (98), with a dorsal-to-ventral distribution of functions related to “vision for action” versus “vision for perception” (99), and a left-to-right distribution of functions related to self-directed motoric and verbal functions (LH) versus bottom-up and/or top-down allocation of attention to external sensory content (RH) [for review, see Ref. (97)].

This left–right dimensionality is evident across superior, inferior, and temporal–parietal regions. For example, the RH superior-parietal lobe (SPL) shows specialization for spatial orienting (99), while the LH SPL shows specialization for self-initiated fine motor actions such as writing (99, 100). Moreover, RH IPL lesions often produce impaired sensory-orienting toward the left half of space (i.e., neglect) (101), while LH IPL lesions often produce inaccurate grasping of objects (i.e., apraxia) (102). The RH IPL has also been associated with maintaining focus over prolong periods, either to detect a rare event against a quiet background (vigilance) or to distinguish target stimuli from a stream of continuously presented items (sustained attention) [for review, see Ref. (97)], while the LH IPL has also been associated with reading (65).

Continuing this pattern of left–right specialization, the RH temporal–parietal junction (TPJ) has been associated with stimulus-driven attentional shifting (103, 104), while LH TPJ has been associated with verbal articulatory coding (i.e., naming) (9). In fact, the RH TPJ is currently a point of interest across many fields (e.g., social, memory, attention, etc.), with each tending toward domain specific understandings of its role [for review, see Ref. (104)]. However, broader conceptualizations have begun to emerge. For instance, Geng and Vossel (104) argued that the RH TPJ plays a general role in maintaining/updating the neural context by which the relevance of incoming sensory information is vetted, with greater activation indexing a more flexible attentional and cognitive set (i.e., with more active updating) and reduced activation indexing a more narrow and fixed attentional and cognitive set (i.e., with less active updating). They also specify that this process is likely to draw on multiple distributed brain systems and integrate both bottom-up and top-down processing. This view importantly suggests that moment-to-moment variability in RH TPJ activation might index the degree to which an individual is oriented toward a more fixed versus flexible attentional and cognitive set.

### Rightward parietal asymmetry and ADHD

The current study demonstrated abnormal increased rightward asymmetry in ADHD subjects across each of these parietal brain regions (SPL/P4–P3, IPL/P8–P7, TPJ/P8–P7). This indicates that, during the CPT, ADHD subjects exhibit some form of increased weighting of right-lateralized brain regions previously associated with: orienting attention in space (RH SPL), sustained attention (RH IPL), and more flexible attention and cognitive sets (RH TPJ). The most robust and perhaps straightforward expression of this outcome is likely indicated by our beta2 findings.

EEG beta has been associated with attention-directed information processing (72–75, 105), and particularly so in parietal brain regions (72, 75, 76, 106, 107). More specifically, it is thought to be associated with mechanisms that potentiate early stage encoding of attentionally selected sensory information [for review, see Ref. (72–74)]. Consistent with this, EEG beta has been shown to track hemispherically specialized operations with leftward biased expression during verbal tasks and rightward biased expression during non-verbal tasks (75, 76). This literature suggests that EEG beta is an appropriate measure to capture variability in lateralized contributions to sensory information processing.

ADHD subjects exhibited rightward beta2 asymmetry in all parietal indices (P4–P3, P8–P7, TP8–TP7) with increased unilateral right-sided beta2 power evident for superior (P4) and inferior (P8) aspects. The relative weakness of the unilateral P8 effect when compared to P8–P7 asymmetry and the lack of unilateral effects in temporal–parietal regions indicates that rightward beta2 asymmetry is the critical metric within inferior and temporal–parietal brain regions. In contrast, the relative strength of the unilateral P4 effect versus P4–P3 asymmetry suggests that greater right-sided beta2 power is the critical metric within superior-parietal regions. Hence, we can refine our description of our findings to say that, during the CPT, ADHD subjects exhibited a relative increased weighting of RH versus LH contribution (as measured by rightward beta2 asymmetry) across inferior and temporal–parietal regions along with greater unilateral RH superior-parietal contribution (as measured by P4 beta2 power). Generally speaking, this pattern suggests some form of abnormal increased weighting of external-perceptual versus verbal-motoric processing across inferior and temporal–parietal brain regions with possible associated abnormalities involving the distribution of attention in space (the RH SPL effect).

ADHD rightward EEG asymmetry was additionally evident in the theta band at the superior-parietal index (P4–P3), and in theta and beta1 bands at the temporal–parietal index (TP8–TP7). EEG theta has been implicated in the coordination of long-range cortical interactions and internally oriented brain functions such as working memory (108). The band we have called beta1 (12–16 Hz), also known as the sensory motor rhythm, has been suggested to play a role in the top-down regulation of motor actions (109). According to these views, our theta findings may indicate some form of abnormal long-range integration of RH versus LH superior and temporal–parietal brain regions, highlighting that abnormal rightward asymmetry in ADHD may be linked to broader difficulties with distributed task-directed brain functions (11). Moreover, the multiple frequency band effects (theta, beta1, beta2) at the TP8–TP7 index may be consistent with the above noted complex attentional control functions that have been linked to the RH TPJ (i.e., updating neural context and regulation of fixed versus flexible attentional sets).

### Correlations among EEG asymmetry indices

As in our previous study, ADHD subjects demonstrated a lack of normal association (i.e., observed in controls) between beta2 asymmetry at the inferior (P8–P7) and temporal–parietal (TP8–TP7) AIs during the CPT. This demonstrates an ADHD reduced coordination of *functional asymmetry* between brain regions thought to support attentional-state setting and applied attentional operations. Coordination of such mechanisms may be a key aspect of successful task-directed brain functioning (11, 110) as possibly demonstrated by the much stronger correlation between beta2 asymmetry at these regions in controls than in ADHD subjects, along with ADHD subjects’ more variable CPT performance. Regardless, this finding makes clear that both abnormal functioning and abnormal coordination of IPL and TPJ brain regions is evident in ADHD subjects during the CPT.

### Asymmetry and cognition

Our analysis of EEG-to-cognition showed a pattern of abnormal reversed association between parietal asymmetry and cognitive abilities in ADHD. That is, where rightward asymmetry was associated with better or worse performance in ADHD subjects, an opposite pattern or non-effect was evident in controls. The majority of these findings indicated that rightward temporal–parietal asymmetry (across multiple frequencies) was associated with better VWM ability in ADHD, but worse VWM ability in controls. There were far fewer cognitive associations with superior (P4–P3: theta and beta2) and inferior (P8–P7: beta2) asymmetry. Asymmetry in these regions was exclusively associated with tasks that required constant attention to external visual stimuli, and for these sensory demanding tasks, ADHD subjects’ rightward superior and inferior parietal asymmetry was exclusively associated with worse performance.

We previously discussed that increased RH TPJ activation occurs with flexible shifting attention. It has also been linked to the use of mental imagery. Greater activation of RH TPJ has been shown to occur during mental rotation tasks (111–114) including mental rotation of numbers and letters (114). Moreover, RH TPJ-induced hemi-neglect has been linked to a reduced capacity to use mental imagery during math operations (115). These findings suggest a possible benefit for flexible shifting attention during the use of mental imagery, possibly reflecting a need to rapidly integrate information across distributed features of mentally constructed images. Consistent with this, social neuroscience studies have highlighted that RH TPJ induced “flexible attention” is adaptive under circumstances that require integrating spatially distributed information (103).

According to this literature, the observed association between rightward TP8–TP7 asymmetry and better VWM ability in ADHD might reflect an ADHD default bias toward flexible attention and visual forms of cognition, which although maladaptive for the CPT, may bear advantages for tasks that benefit from mental imagery. ADHD subjects did exhibit better performance on the backward digit span task, which of the tasks utilized, is arguably the most likely to benefit from mental imagery. Moreover, in ADHD subjects only, rightward frontal beta2 asymmetry (F8–F7) was associated with better mental arithmetic, and our own and others’ previous work (described in the introduction) has indicated greater reliance on, or bias toward, visual cognition and sensory processing strategies in ADHD (12–15, 59).

Finally, as noted, rightward superior (P4–P3) and inferior (P8–P7) parietal asymmetry in ADHD was exclusively associated with worse performance for tasks that required continuous attention to external visual stimuli and minimal internal computation. One interesting possibility is that poor task-regulation of RH TPJ function may create a circumstance, whereby ADHD individuals must continually reassert compensatory attention following task-disruptive attentional shifting during the CPT. If true, both rightward asymmetry at superior and inferior parietal indices, and their negative association to sensory weighted behavioral tasks, may reflect ADHD subjects’ attempting to regain their “attentional footing” (i.e., compensatory applied attention) following task-disruptive attentional shifts.

### Summary and conclusion

The current study replicated our previous finding showing that ADHD adults exhibit rightward inferior parietal (P8–P7) beta2 asymmetry during the CPT. Additional novel analyses established that this was part of a broader pattern of abnormal rightward beta2 asymmetry in superior (P4–P3), inferior (P8–P7), and temporal–parietal (TP8–TP7) regions, with additional frequency bands effects for the temporal–parietal (theta, beta1) and superior-parietal (theta) indices. We also replicated our previous finding showing a reduced association between P8–P7 and TP8–TP7 beta2 asymmetry in ADHD. Finally, novel analyses indicated that rightward TP8–TP7 asymmetry in ADHD was associated with better VWM ability, while rightward P8–P7 and P4–P3 asymmetry was associated with worse performance for task requiring continuous attention to visual stimuli.

In considering this pattern of results, we suggest a bipartite view of right biased parietal brain function in ADHD that highlights two semi-independent mechanisms, one linked to general state setting operations (RH TPJ), and one linked to applied attention during task-operations (RH IPL). We expect that ADHD individuals may exhibit variability in the primacy and strength of such effects, and/or with regards to their task-adaptive coordination. However, regardless of such variability, we expect that poor functioning and/or coordination of these mechanisms likely results in a convergent requirement for compensatory applied (i.e., sustained/selective) attention during the CPT, which may explain the relative statistical robustness of the rightward P8–P7 beta2 asymmetry finding. This speculation also suggests that rightward P8–P7 beta2 asymmetry and possibly reduced coordination of P8–P7 and TP8–TP7 beta2 asymmetry may generally index poor task-directed brain function in ADHD (i.e., a convergent deficit effect) (11).

### Additional considerations

The notion of abnormal functioning and/or coordination of both applied and state setting attentional operations in ADHD is well aligned with several recognized domains of ADHD pathology. For instance, abnormal norepinephrine (NE) function is implicated in ADHD (116), and top-down regulation of the brain-stem locus coeruleus, which is the primary source of dense NE projections to the RH parietal cortex (117), is critical for shifting between fixed and flexible attentional states (118, 119). Hence, this system may be relevant to the discussed attentional-state setting aspects of rightward parietal asymmetry in ADHD.

Furthermore, ADHD has been associated with abnormal network functioning (default mode, dorsal and ventral attention, and fronto-parietal), which implicates abnormal parietal brain function (58, 120). Although the default mode network (DMN) was previously considered as a task-negative system (121), it is now understood to play a role in self-referential aspects of cognition (122) and internal aspects of task-directed brain function (123, 124), including VWM (125). There are even indications that the DMN plays a regulatory role over task-positive networks (126). Consistent with this, medial frontal and orbitofrontal brain regions linked to the DMN (127) are apparent sources of top-down regulation of the brain-stem locus coeruleus, which as noted, is critical for regulating transitions between controlled and flexible attentional and cognitive states (118, 119). It is interesting to consider that ADHD abnormal DMN function might undermine the coordination of additional task-positive networks, and thus broadly impact task-directed brain functions, including task-directed visual sensory information processing (11).

Rightward parietal EEG asymmetry in ADHD is also well aligned with identified ADHD reduced posterior corpus callosum size (42, 128) and function (34, 43–45). The specific region implicated (the splenium) connects left and right visual and parietal cortices (129) and undergoes increases of myelination across development that are coincident with a growing capacity to regulate lateralized visual functions (130). These changes include a progression from right-to-left dominance of visual operations (130–132). Hence, ADHD abnormal rightward parietal asymmetry might reflect some form of deviant maturation of callosal functioning that bears on interhemispheric coordination of visual operations, possibly resulting in greater RH contribution. Perhaps consistent with this, ADHD has been associated with atypical faster left-to-right colossal transfer times (45), larger RH visual cortical volumes (41), enhanced ability to inhibit pre-potent LH based stimulus responsivity (12), and slow verbal naming speeds (20–27).

### Limitations

Although replication of our EoI was highly significant in our initial (*p* = 0.00001) and full sample (*p* = 0.00003) and a pattern of rightward asymmetry was evident across all parietal findings, this effect was weaker in our intermediate sample alone (i.e., new sample of 43 ADHD individuals: *p* = 0.004, and with medication adjustment: *p* = 0.046). There are possible methodological reasons for this, such as unidentified variation in recruitment strategies, EEG time of day, and/or technician effects over the course of the study (approximately 3 years). However, it is also possible that variability in the strength of this effect portends key information.

We have recently presented a model of ADHD abnormal brain function (11), which operationalizes ADHD as poor functioning of a distributed set of brain systems that get dynamically integrated in service to complex task-directed actions (see Introduction). The model supposes that any degradation to this system’s operational capacity (i.e., no matter the cause) results in less-efficient task-directed control over visual sensory encoding, with associated increased RH contributions tied to greater processing of task-extraneous visual content and/or compensatory attentional effort.

This framework suggests that atypical rightward asymmetry should be broadly reflective of any form of non-optimized task-directed brain functioning, and consistent with this, greater RH contribution to sensory processing has been reported across multiple circumstances linked to attention difficulties and that are often comorbid with ADHD (e.g., anxiety, depression, sleep deprivation, novelty seeking, reading disability, etc.) (60–70), as well as ADHD risk factors, such as left handedness (133) and being male (134).

The model also importantly suggests that rightward parietal asymmetry in ADHD is likely to manifest along a continuum of severity that reflects different classes of underlying causal mechanisms, i.e., from fixed modular deficits, to more subtle brain-state derived issues. The currently utilized CPT requires prolonged maintenance of a task-oriented state under conditions of low-reward and is perhaps well suited to capture ADHD asymmetry effects even in the face of such variability. However, optimal assessment of rightward parietal asymmetry in ADHD may generally require time-extended within subject assessment and/or large *n*-sizes, as was used in our current full sample. In short, we expect that the weaker rightward P8–P7 beta2 asymmetry uncovered in our intermediate ADHD sample may reflect natural variability associated with this effect. We also expect that such variability likely underlies the observed small-to-moderate effect sizes that were associated with the reported group differences in parietal asymmetry.

There are several additional limitations and/or circumstance that should be considered. First, all subjects in the current study, ADHD and controls, were the biological parents of children with ADHD. This bears the possibility that both groups may represent unique variants of adult ADHD and control samples. The ADHD group may reflect a particularly heritable ADHD variant, while controls may represent a unique group inclined to marry ADHD individuals and/or who are perhaps carriers of subclinical ADHD qualities. If the latter is true, this may have reduced our ability to detect group differences.

Next, EEG involves multiple sources of measurement (electrodes) with each producing multiple data components (multi-hertz signal), making the issue of multiple testing a central challenge. Researchers sometimes average signal across electrodes to limit the number of tests, but this sacrifices analytic resolution. Another approach, repeated measures ANOVA bears data loss issues as data must be available for each level of every included variable in order for a given subject’s data to be utilized, and because of channel and/or frequency specific EEG artifact this is a significant issue. Linear multilevel modeling can circumvent this problem; however, this approach was not utilized in the original study that we sought to replicate.

The original study used univariate ANOVA to test group differences in individual AIs, and due to our specific interest in replicating that study’s finding we used the same approach. This results in many analyses being performed increasing the risk of type-1 error. However, we judged that the highly targeted nature of our replication analyses and our specific *a priori* hypothesis of rightward parietal asymmetry in ADHD strongly limits this risk, and so we did not correct for multiple testing. Moreover, with the expanded analyses our primary goal was to identify coherent *patterns* of results (in EEG asymmetry and EEG asymmetry association to cognition), which also reduces the risk of type-1 error. In short, we acknowledge that without correcting for multiple testing our current results remain vulnerable to type-1 error. However, given the clear patterns within our current findings and their alignment with our earlier study results and our *a priori* hypothesis, we feel this possibility is remote.

Lastly, the original study required a current diagnosis of ADHD, whereas this study used a lifetime diagnosis. A lifetime diagnosis may include individuals who had childhood ADHD that waned as they grew into adults. In our current full sample, this represented 23 of 90 ADHD subjects (i.e., 67 had current ADHD diagnoses). The decision to include ADHD individuals based on a lifetime diagnosis reflects a growing awareness that non-persistent ADHD may be an important variant form of adult-compensated rather than adult-remediated ADHD. That is, recent findings suggest that while some ADHD individuals grow to compensate for and/or experience diminished clinical symptoms, their brain functioning and cognitive abilities may continue to exhibit atypical ADHD-linked characteristics (16, 135, 136). Furthermore, in order to establish whether rightward parietal EEG asymmetry is a durable and general feature of ADHD, we thought it best to try to demonstrate this characteristic using the broad lifetime based description of the disorder.

## Conflict of Interest Statement

The authors declare that the research was conducted in the absence of any commercial or financial relationships that could be construed as a potential conflict of interest.
